# Diethyl pyrazine-2,5-dicarboxyl­ate

**DOI:** 10.1107/S1600536812028164

**Published:** 2012-06-27

**Authors:** Chuan-Hui Li, Wen-Shi Wu, Yu-Min Huang

**Affiliations:** a College of Materials Science and Engineering, Huaqiao University, Xiamen, Fujian 361021, People’s Republic of China

## Abstract

The mol­ecule of the title compound, C_10_H_12_N_2_O_4_, is located around an inversion center. The carboxyl­ate groups are twisted slightly with respect to the pyrazine ring, making a dihedral angle of 2.76 (19)°. In the crystal, mol­ecules are stacked along the *c* axis *via* weak C—H⋯O hydrogen bonds.

## Related literature

For the structures of related compounds, see: Zhang *et al.* (2010 ▶); Cockriel *et al.* (2008 ▶).
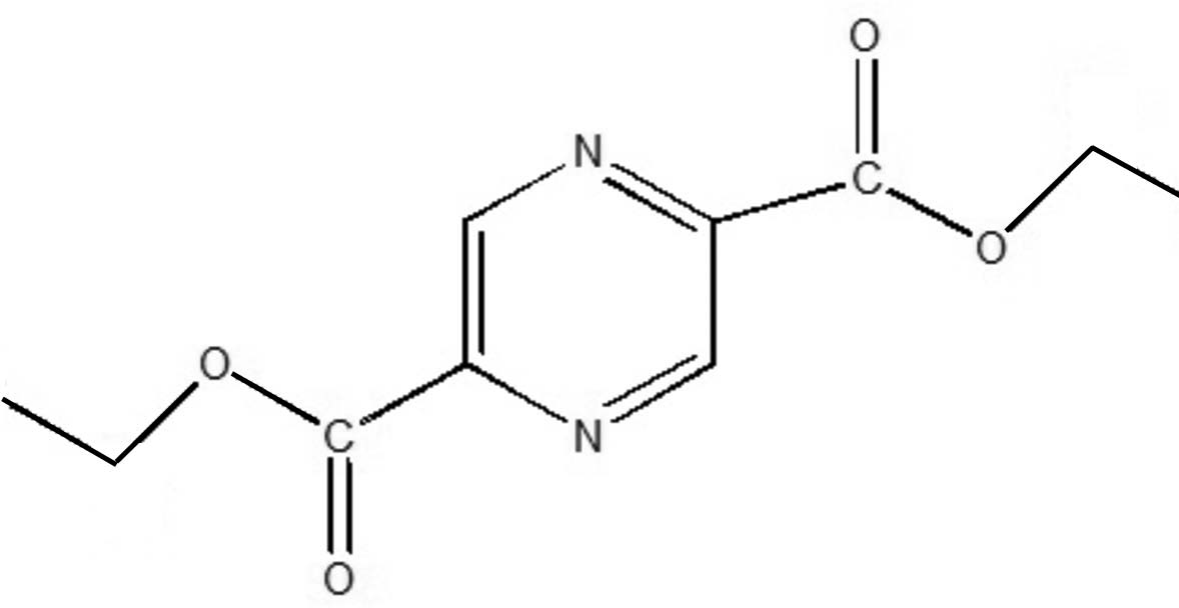


## Experimental

### 

#### Crystal data

C_10_H_12_N_2_O_4_
*M* *_r_* = 224.22
Monoclinic, 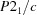

*a* = 12.284 (6) Å
*b* = 5.640 (3) Å
*c* = 7.881 (4) Å
β = 108.713 (9)°
*V* = 517.2 (5) Å^3^
*Z* = 2
Mo *K*α radiation
μ = 0.11 mm^−1^
*T* = 173 K
0.7 × 0.3 × 0.05 mm

#### Data collection

Bruker SMART diffractometer
Absorption correction: multi-scan (*SADABS*; Sheldrick, 1996 ▶) *T*_min_ = 0.960, *T*_max_ = 0.994
2994 measured reflections
1317 independent reflections
1055 reflections with *I* > 2σ(*I*)
*R*_int_ = 0.031

#### Refinement

*R*[*F* ^2^ > 2σ(*F* ^2^)] = 0.053
*wR*(*F* ^2^) = 0.131
*S* = 1.07
1317 reflections
73 parameters
H-atom parameters constrained
Δρ_max_ = 0.21 e Å^−3^
Δρ_min_ = −0.31 e Å^−3^

### 

Data collection: *SMART* (Bruker, 1997 ▶); cell refinement: *SAINT* (Bruker, 1999 ▶); data reduction: *SAINT*; program(s) used to solve structure: *SHELXS97* (Sheldrick, 2008 ▶); program(s) used to refine structure: *SHELXL97* (Sheldrick, 2008 ▶); molecular graphics: *SHELXTL* (Sheldrick, 2008 ▶); software used to prepare material for publication: *SHELXTL*.

## Supplementary Material

Crystal structure: contains datablock(s) I, global. DOI: 10.1107/S1600536812028164/is5143sup1.cif

Structure factors: contains datablock(s) I. DOI: 10.1107/S1600536812028164/is5143Isup2.hkl

Supplementary material file. DOI: 10.1107/S1600536812028164/is5143Isup3.cml

Additional supplementary materials:  crystallographic information; 3D view; checkCIF report

## Figures and Tables

**Table 1:** Hydrogen-bond geometry (Å, °)

*D*—H⋯*A*	*D*—H	H⋯*A*	*D*⋯*A*	*D*—H⋯*A*
C4—H4*A*⋯O2^i^	0.97	2.58	3.537 (3)	168

Symmetry code: (i) 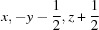.

**Fig. 1.**
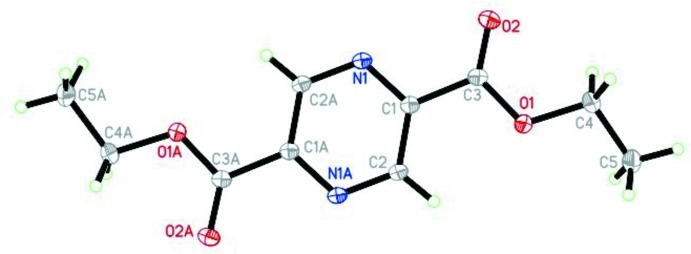
The molecular structure (at 30% probability) of the title compound. [Symmetry code: (A) 1 - x, -y, 1 - z].

**Fig. 2.**
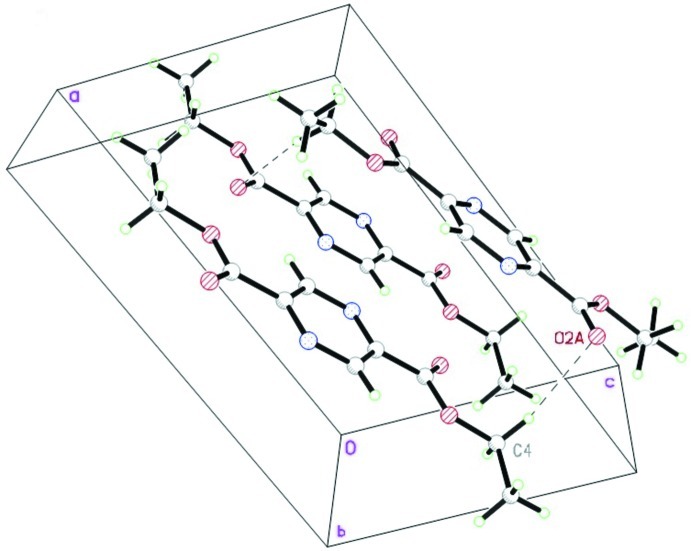
Packing diagram of the title complex, showing hydrogen bonds as dashed lines. [Symmetry code: (A) x, -0.5 - y, 0.5 + z].

## supplementary crystallographic information

### Comment

The structure of the title compound is illustrated in Fig. 1. The molecule of title compound, C_10_H_12_N_2_O_4_, is essentially planar and the carboxylate groups are twisted slightly with respect to the pyrazine ring, making a dihedral angle of 2.76 (19)°. The carboxyl C—O and C═O bonds are normal, while the bond angle of C—N═C are slightly larger than those in diisopropyl pyrazine-2,5-dicarboxylate (Zhang *et al.*, 2010). The angle C3—O1—C4 of 116.05° is larger compared to the value of 115.05° in pyrazine-2,5-dicarboxylic acid dimethyl ester (Cockriel *et al.*, 2008). The crystal structure is stabilized *via* van der Waals forces and week C—H···O hydrogen bonds (Fig. 2 and Table 1).

### Experimental

The title compound was synthesized by dissolving 2,5-pyrazinedicarboxylic acid (2 g, 11.9 mmol) in 200 ml ethanol, while stirring 2 ml concentrated H_2_SO_4_ was added slowly. The solution was left to reflux for 12 h, then distillation under reduced pressure until no solution to outflow. The solution was made neutral with Na_2_CO_3_(aq), extracted with 30 ml ethyl acetate. Transparent crystals of the title compound were obtained by slow evaporation at room temperature for ten days.

### Refinement

H atoms were included in a riding model approximation with C—H = 0.93–0.97 Å, and with *U*_iso_(H) = 1.2*U*_eq_(C) or 1.5*U*_eq_(C_mehtyl_) .

### Crystal data

**Table:**

C_10_H_12_N_2_O_4_	*F*(000) = 236.0
*M**_r_* = 224.22	*D*_x_ = 1.440 Mg m^−^^3^
Monoclinic, *P*2_1_/*c*	Mo *K*α radiation, λ = 0.71073 Å
Hall symbol: -P 2ybc	Cell parameters from 2994 reflections
*a* = 12.284 (6) Å	θ = 4.0–28.5°
*b* = 5.640 (3) Å	µ = 0.11 mm^−^^1^
*c* = 7.881 (4) Å	*T* = 173 K
β = 108.713 (9)°	Plate, colourless
*V* = 517.2 (5) Å^3^	0.7 × 0.3 × 0.05 mm
*Z* = 2	

### Data collection

**Table:**

Bruker SMART diffractometer	1317 independent reflections
Radiation source: fine-focus sealed tube	1055 reflections with *I* > 2σ(*I*)
Graphite monochromator	*R*_int_ = 0.031
ω scans	θ_max_ = 28.5°, θ_min_ = 4.0°
Absorption correction: multi-scan (*SADABS*; Sheldrick,1996)	*h* = −16→14
*T*_min_ = 0.960, *T*_max_ = 0.994	*k* = −5→7
2994 measured reflections	*l* = −10→10

### Refinement

**Table:**

Refinement on *F*^2^	Primary atom site location: structure-invariant direct methods
Least-squares matrix: full	Secondary atom site location: difference Fourier map
*R*[*F*^2^ > 2σ(*F*^2^)] = 0.053	Hydrogen site location: inferred from neighbouring sites
*wR*(*F*^2^) = 0.131	H-atom parameters constrained
*S* = 1.07	*w* = 1/[σ^2^(*F*_o_^2^) + (0.0606*P*)^2^ + 0.1472*P*] where *P* = (*F*_o_^2^ + 2*F*_c_^2^)/3
1317 reflections	(Δ/σ)_max_ < 0.001
73 parameters	Δρ_max_ = 0.21 e Å^−^^3^
0 restraints	Δρ_min_ = −0.31 e Å^−^^3^

### Special details

**Table:**

Geometry. All e.s.d.'s (except the e.s.d. in the dihedral angle between two l.s. planes) are estimated using the full covariance matrix. The cell e.s.d.'s are taken into account individually in the estimation of e.s.d.'s in distances, angles and torsion angles; correlations between e.s.d.'s in cell parameters are only used when they are defined by crystal symmetry. An approximate (isotropic) treatment of cell e.s.d.'s is used for estimating e.s.d.'s involving l.s. planes.
Refinement. Refinement of *F*^2^ against ALL reflections. The weighted *R*-factor *wR* and goodness of fit *S* are based on *F*^2^, conventional *R*-factors *R* are based on *F*, with *F* set to zero for negative *F*^2^. The threshold expression of *F*^2^ > σ(*F*^2^) is used only for calculating *R*-factors(gt) *etc*. and is not relevant to the choice of reflections for refinement. *R*-factors based on *F*^2^ are statistically about twice as large as those based on *F*, and *R*- factors based on ALL data will be even larger.

### Fractional atomic coordinates and isotropic or equivalent isotropic displacement parameters (Å^2^)

**Table:**

	*x*	*y*	*z*	*U*_iso_*/*U*_eq_	
N1	0.46961 (11)	−0.2315 (2)	0.52651 (18)	0.0275 (3)	
O1	0.26007 (9)	0.0921 (2)	0.64425 (16)	0.0308 (3)	
O2	0.28072 (11)	−0.3005 (2)	0.63126 (19)	0.0401 (4)	
C1	0.41208 (12)	−0.0450 (3)	0.55512 (19)	0.0241 (3)	
C2	0.44174 (13)	0.1860 (3)	0.5289 (2)	0.0265 (4)	
H2A	0.3986	0.3110	0.5507	0.032*	
C3	0.31162 (13)	−0.1030 (3)	0.6147 (2)	0.0265 (4)	
C4	0.16106 (15)	0.0568 (3)	0.7018 (3)	0.0359 (4)	
H4A	0.1848	0.0010	0.8247	0.043*	
H4B	0.1102	−0.0605	0.6266	0.043*	
C5	0.10095 (15)	0.2883 (3)	0.6871 (2)	0.0377 (4)	
H5A	0.0725	0.3356	0.5636	0.057*	
H5B	0.1537	0.4058	0.7549	0.057*	
H5C	0.0378	0.2732	0.7333	0.057*	

### Atomic displacement parameters (Å^2^)

**Table:**

	*U*^11^	*U*^22^	*U*^33^	*U*^12^	*U*^13^	*U*^23^
N1	0.0314 (7)	0.0174 (6)	0.0369 (7)	−0.0001 (5)	0.0154 (6)	0.0002 (5)
O1	0.0289 (6)	0.0234 (6)	0.0457 (7)	0.0012 (4)	0.0199 (5)	0.0006 (5)
O2	0.0450 (8)	0.0234 (7)	0.0617 (9)	−0.0062 (5)	0.0307 (6)	−0.0005 (6)
C1	0.0256 (7)	0.0211 (8)	0.0256 (7)	−0.0009 (6)	0.0080 (6)	−0.0005 (6)
C2	0.0304 (8)	0.0184 (7)	0.0328 (8)	0.0014 (6)	0.0132 (6)	0.0000 (6)
C3	0.0284 (8)	0.0212 (8)	0.0304 (8)	−0.0010 (6)	0.0104 (6)	0.0007 (6)
C4	0.0330 (9)	0.0337 (9)	0.0493 (10)	0.0000 (7)	0.0247 (8)	0.0048 (8)
C5	0.0330 (9)	0.0410 (11)	0.0450 (10)	0.0046 (7)	0.0208 (8)	0.0018 (8)

### Geometric parameters (Å, º)

**Table:**

N1—C2^i^	1.322 (2)	C2—H2A	0.9300
N1—C1	1.326 (2)	C4—C5	1.486 (3)
O1—C3	1.3270 (19)	C4—H4A	0.9700
O1—C4	1.442 (2)	C4—H4B	0.9700
O2—C3	1.197 (2)	C5—H5A	0.9600
C1—C2	1.386 (2)	C5—H5B	0.9600
C1—C3	1.490 (2)	C5—H5C	0.9600
			
C2^i^—N1—C1	116.31 (14)	O1—C4—H4A	110.2
C3—O1—C4	116.04 (13)	C5—C4—H4A	110.2
N1—C1—C2	122.69 (14)	O1—C4—H4B	110.2
N1—C1—C3	114.83 (14)	C5—C4—H4B	110.2
C2—C1—C3	122.47 (14)	H4A—C4—H4B	108.5
N1^i^—C2—C1	121.00 (15)	C4—C5—H5A	109.5
N1^i^—C2—H2A	119.5	C4—C5—H5B	109.5
C1—C2—H2A	119.5	H5A—C5—H5B	109.5
O2—C3—O1	124.49 (15)	C4—C5—H5C	109.5
O2—C3—C1	124.19 (15)	H5A—C5—H5C	109.5
O1—C3—C1	111.31 (13)	H5B—C5—H5C	109.5
O1—C4—C5	107.59 (14)		
			
C2^i^—N1—C1—C2	0.1 (3)	N1—C1—C3—O2	−2.3 (2)
C2^i^—N1—C1—C3	179.07 (13)	C2—C1—C3—O2	176.70 (16)
N1—C1—C2—N1^i^	−0.1 (3)	N1—C1—C3—O1	178.63 (13)
C3—C1—C2—N1^i^	−178.99 (14)	C2—C1—C3—O1	−2.4 (2)
C4—O1—C3—O2	0.6 (2)	C3—O1—C4—C5	−167.24 (14)
C4—O1—C3—C1	179.71 (13)		

Symmetry code: (i) −*x*+1, −*y*, −*z*+1.

### Hydrogen-bond geometry (Å, º)

**Table:**

*D*—H···*A*	*D*—H	H···*A*	*D*···*A*	*D*—H···*A*
C4—H4*A*···O2^ii^	0.97	2.58	3.537 (3)	168

Symmetry code: (ii) *x*, −*y*−1/2, *z*+1/2.
